# Anthropometric study of the scapula in a contemporary population from granada. Sex estimation and glenohumeral osteoarthritis prevalence

**DOI:** 10.1371/journal.pone.0305410

**Published:** 2024-07-10

**Authors:** Adoración Garzón-Alfaro, Miguel Botella, Guillermo Rus Carlborg, Nicolás Prados Olleta, Amanda Rocío González- Ramírez, Pedro Hernández-Cortés

**Affiliations:** 1 Orthopedic Surgery Department, Upper Limb Surgery Unit, “San Cecilio” University Hospital of Granada, Madrid, Spain; 2 Department of Anthropology, School of Medicine of Granada, Madrid, Spain; 3 Department of Structural Mechanics, Ultrasonics Group (TEP-959), University of Granada, Granada, Spain; 4 Excellence Research Unit “ModelingNature” MNat UCE.PP2017.03, University of Granada, Granada, Spain; 5 Biosanitary Research Institute of Granada (IBS), Granada, Spain; 6 Orthopedic Surgery Department, Foot and Ankle Surgery Unit, “Virgen de las Nieves”, University Hospital of Granada, Madrid, Spain; 7 Surgery Department, School of Medicine. Granada University, Granada, Spain; 8 Bio- Health Research Foundation of Eastern Andalusia- Alejandro Otero (FIBAO), Granada, Spain; AIIMS: All India Institute of Medical Sciences, INDIA

## Abstract

Anthropometric studies of the scapula have been rare in Spanish populations, nevertheless they are of current interest in forensic anthropology for estimation of sex. Although the estimation of sex is usually carried out on the pelvis and skull, other measurements related to the scapula can be helpful when the skeletal remains are incomplete. Glenohumeral osteoarthritis development is influenced, among others, by the morphology of the scapula, which is one of the less studied aspects. We carried out a descriptive study of anthropometric parameters in a series of 157 scapulae (82 individuals) on bone remains dated to the 20^th^ century from a population of Granada (Southern Spain). Seventy seven (49%) were right-side and 80 (51%) left-side; 72 (45.9%) were from males and 85 (54.1%) from females, and the mean age at death was 70.76±11.7 years. The objective was to develop a discrimination function for sex estimation based on anthropometric parameters of the scapula other than those considered to date, and to analyze the prevalence of glenohumeral osteoarthritis in relation to selected anthropometric parameters. A logistic regression model based on parameters of the upper-external segment of the scapula was done. The obtained formula: 1/1+e^ (- (-57.911 + 0.350*B + 0283*C + 0.249*b + 0.166*a +-0.100*β) classifies male sex with 98.3% accuracy and female sex with 92.1%. Glenohumeral osteoarthritis was detected in 16.6% of individuals and was related to age (p<0.05), scapular length (p<0.05), glenoid width (p<0.05), glenopolar angle (p<0.05), and α angle (p<0.05) in bivariate analyses but showed no significant associations in multivariate analyses. This approach can be useful for anthropological-forensic identification when scapula remains are incomplete. Glenohumeral osteoarthritis is significantly associated with a smaller α angle.

## Introduction

The scapula connects the upper extremity to the axial skeleton. This triangular-shaped bone is in the posterolateral area of the thorax and forms, along with the humerus, the shoulder joint. Its shape is influenced by the constitutional morphology of the bone and the postnatal pattern of skeletal growth [1], activities undertaken, corresponding adaptations of the musculoskeletal system [2], and the aging process [3], among other factors.

The pelvis and skull are conventionally used for sex estimation from bone remains in the forensic medical setting. However, a large amount of this information is often lost due to taphonomic processes, and other bones must be used for sex estimation [4–6].

The scapula has served to determine sex in anthropological-forensic settings [4, 7], and various discrimination functions have been developed [4, 7–13]. Notably, the function applied to the “Cretan collection”, based on glenoid fossa width and maximum scapular spine length, proved able to determine the sex of individuals with 95.9% accuracy [10]. Postmortem CT measurements of scapulae, analyzed using logistic discriminant function developed in the study of [13], showed 94.5% accuracy in estimating sex.

The osteoarticular disease that more often affects the scapula is glenohumeral joint arthrosis, which can be detected in bone remains [14–19]. It has been estimated to have a prevalence of 17% among the over-65-year-old european population [20] and is usually secondary to degenerative rotator cuff tendon tears [19]. However, development of this disease is influenced by multiple factors, including the morphology of the scapula [18], one of the less well studied aspects. Recently, a small critical shoulder angle (CSA) was found to be associated with a high prevalence of glenohumeral osteoarthritis [14, 15, 17] and the acromion glenoid angle correlates with the development of rotator cuff tear and glenohumeral osteoarthritis too [16].

Anthropometric studies of the scapula have been undertaken in various populations but have been rare in Spanish populations [21], especially in Andalusian populations [22].

The objectives of the present anthropological study of scapulae from a contemporary population of Granada are: 1) to develop a discrimination function for sex estimation in this population based on anthropometric parameters of the scapula other than those previously considered for sex identification, allowing sex estimation from incomplete scapulae lacking known reference points, and 2) to determine the prevalence of glenoid joint degeneration and its association with selected anthropometric parameters.

## Materials and methods

This descriptive study of anthropometric parameters of scapulae was conducted in bone remains from San José cemetery [23] in Granada (Spain). It is a large and expanding osteological collection of identified individuals of all ages from the Granada municipal cemetery (San Jose). It was started in 1991, when the San Jose cemetery board granted our Laboratory of Anthropology permission to hold and investigate bone remains that were otherwise destined for incineration or interment in a communal plot. This collection is housed in the Laboratory of Anthropology of the University of Granada, Spain. It mainly dates from the mid-20th century. The state of preservation is very good, and antemortem information is available from burial and death certificates, among other documents. The protocol for studying these bone remains was approved by the ethics committee of the University of Granada and complies strictly with national regulations (Law 14/2007, 3 July) on the protection of personal privacy and confidential treatment of personal data in biomedical research.

This study included 125 randomly selected skeletons.

### Inclusion and exclusion criteria

The inclusion criteria for scapulae were to be complete and in an adequate condition for performing all anthropometric measurements under study. Exclusion criteria for skeletons were immaturity of the individual and evidence of traumatic sequelae or tumor disease. When the two scapulae of the same individual were present, both were included and analysed for the study.

### Series

The final study series comprised 157 scapulae (82 individuals; 38 males and 44 females), 77 (49%) right-side and 80 (51%) left-side; 72 (45.9%) were from males and 85 (54.1%) from females, and the mean age at death was 70.76±11.7 years (range, 30–93 years). Biological sex and date of death were documented according to the death certificate.

### Instruments and measurements

**Fig 1:**
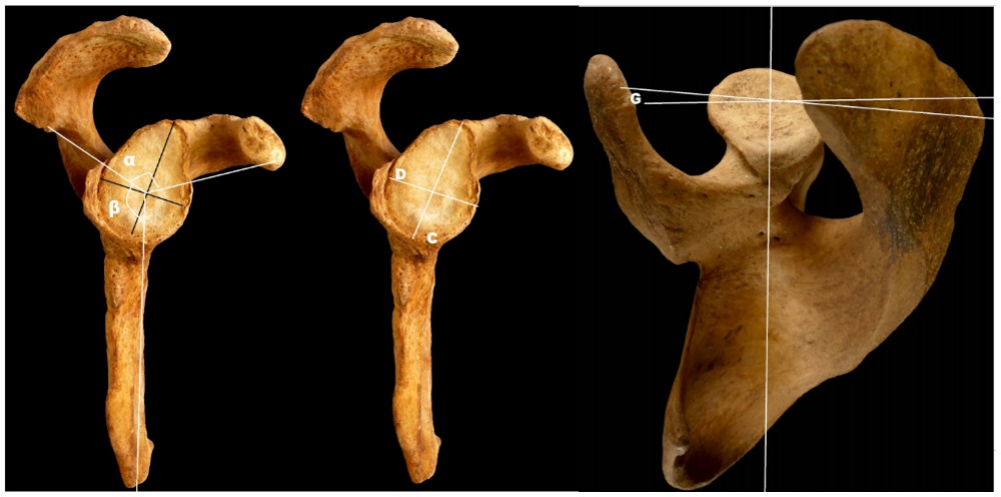
Anthropometric parameters in lateral and superior views. **C: Glenoid height: distance between supraglenoid tubercle and infraglenoid tubercle** [24]D: Glenoid width: major transversal axis perpendicular to the glenoid height axis [24] G: Glenoid version: anteversion or retroversion with respect to the horizontal axis of the scapula. Degrees of anteversion or retroversion α: α angle: formed by the lines that connect the center of the glenoid fossa with the tip of the coracoid process and the most posterior point of the acromion [25] β: β angle: formed between the axis of the outer border of the scapula and the line connecting the center of the glenoid fossa to the posterior angle of the acromion.

**Fig 2:**
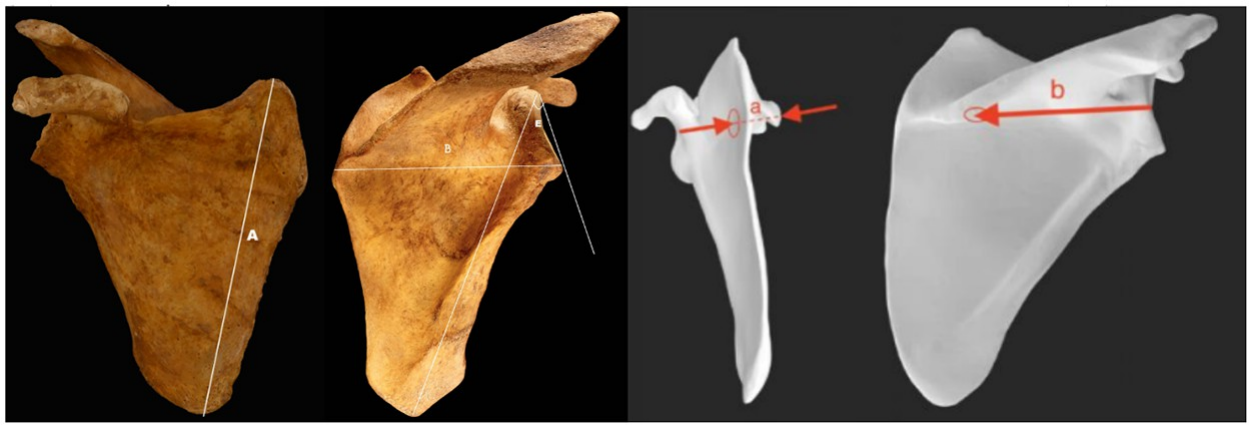
Anthropometric parameters in medial, anterior, and posterior views. A: Scapular length: distance between superior and inferior angle [24] B: Scapular width: distance from vertebral border at scapular spine level to the lowest point of the glenoid fossa [24] E: Glenopolar angle: angle between the line tangent to the articular surface of the glenoid fossa and the line connecting the most cranial point of the glenoid fossa with the lower vortex of the scapula [25] a: Measurement “a”: distance between the deepest area of the anterior face of the scapula and the most dorsal point of the acromion b: Measurement “b”: distance between the posterior edge of the glenoid fossa and the medial tubercle of the scapular spine.

Measurements (in mm and sexagesimal degrees) were performed manually using digital scale rulers, digital goniometers, and the Angle Meter application (smarttoolfactory@icloud.com, Istanbul). The researchers were blinded about any information of gender, age, height or medical history before anthropometric measures were taken. Table 1 and Figs 1 and 2 exhibit the measured parameters.

**Table 1:** Analysed anthropometric parameters.

CODE	PARAMETER	DESCRIPTION
A	Scapular length	Distance measured between the superior and inferior angle
B	Scapular width	Distance from the vertebral border at scapular spine level to the lowest point of the glenoid fossa
C	Glenoid height	Distance between the supraglenoid tubercle and infraglenoid tubercle
D	Glenoid width	Major transversal axis perpendicular to the glenoid height axis
E	Glenopolar angle	Between the line tangent to the articular surface of the glenoid fossa and the line connecting the most cranial point of the glenoid fossa to the lower vortex of the scapula
G	Glenoid version	Anteversion or retroversion with respect to the horizontal axis of the scapula. Degrees of anteroversion or retroversion
a	Measurement “a”	Distance between deepest area of the anterior surface of the scapula and most dorsal point of the acromion
b	Measurement “b”	Distance between posterior edge of glenoid fossa and medial tubercle of scapular spine
α	α angle	Formed by the lines connecting the center of the glenoid fossa and the tip of the coracoid process and the most posterior point of the acromion
β	β angle	Between axis of the outer border of the scapula with the line connecting the center of the glenoid fossa to the posterior angle of the acromion
DA	Glenohumeral osteoarthritis	Presence of degenerative changes: geodes, osteophytes, and/or erosions

Similar studies previously considered the scapular length [24], scapular width [24], glenoid height [24], glenoid width [24] glenopolar angle [25], glenoid version [25], and coracoacromial arch (α angle)[18]. In the present study, distance from the suprascapular notch, measurement “a”, measurement “b”, and measurement β angle were proposed by the authors and introduced into the model for the first time, with the aim of providing alternative measurements in case of incomplete bone remains. The unpublished measurement points were proposed after a pilot study (unpublished data) where anatomical references for shoulder prosthetic surgery were identified. All these variables are continuous and quantitative.

Glenohumeral osteoarthritis was considered when the presence of degenerative changes as geodes, osteophytes, and/or erosions were seen.

### Statistical analysis

SPSS v25.o (IBM SPSS Inc., Armonk, NY) was used for statistical analyses. The normality of the quantitative variables distribution were checked with the Kolmogorov Smirnoff test. The Student’s t-test was used to compare means, considering an alpha error of 5%. Variables that were significant (p≤0.05) in bivariate analysis were entered in multivariate binary logistic regression analysis, including sex and glenohumeral osteoarthritis as dichotomous dependent variables and accepting the same level of significance.

## Results

The distribution of individuals by age was: 21 individuals (13.4%) aged <60 years, 71 (45.2%) 60–75 years, and 46 (29.3%) >75 years. The glenoid was anteverted in 52 (33.3%) scapulae, retroverted in 98 (62.8%), and neutral in 6 (3.8%). The mean anteversion angle was 5.26°±3.59° (1°- 19°) and mean retroversion angle -5.88°±3.82 (-18°-(-1°). Degenerative changes were observed in 26 scapulae (16.6%). Table 2.

**Table 2:** Distribution of individuals of the series.

		MALE	FEMALE
**AGED**	**<60 years**	15 (20.8%)	6 (7.1%)
	**60–75 years**	40 (55.6%)	31 (36.5%)
	**>75 years**	8 (11.1%)	38 (44.7%)
**VERSION**	**Anteverted**	22 (30.6%)	30 (35.3%)
	**Retroverted**	46 (63.9%)	52 (61.2%)
	**Neutral**	3 (4.2%)	3 (3.5%)
**DEGENERATIVE CHANGES**		3 (4.2%)	7 (8.2%)

### Bivariate analysis

Statistically significant differences were observed between sexes in nine anthropometric variables: scapular length, scapular width, suprascapular notch distance, glenoid height, glenoid width, distance “a”, distance “b”, α angle, and β angle. In comparison to females, the mean scapular length was 16 mm higher, the mean scapular width 11 mm higher, the suprascapular notch distance 3 mm longer, mean glenoid height and width both 3 mm higher, mean measurement “a” 6 mm longer, mean measurement “b” 10 mm higher, mean angle α 9° higher, and mean β angle 7° lower in males. All differences were statistically significant (Table 3).

**Table 3:** Bivariate analysis as a function of sex. Student’s t-test.

VARIABLE	MALE (MEAN)	MALE (SD)	FEMALE (MEAN)	FEMALE (SD)	SIGNIFICANCE
A	152.85	16.77	136.72	9.64	≤0.001*
B	103.53	4.57	92.03	6.16	≤0.001*
C	34.45	2.63	31.10	2.42	≤0.001*
D	26.00	2.15	23.08	2.33	≤0.001*
E	42.96	5.17	43.85	5.12	≤0.001*
G	28.46	2.31	25.73	1.77	≤0.001*
a	62.43	5.36	56.73	5.11	≤0.001*
b	70.32	7.38	62.43	5.36	≤0.001*
α	113.74	15.46	104.76	15.27	≤0.001*
β	120.42	13.19	127.91	10.58	≤0.001*

P ≤ 0.05

Significant differences were found between scapulae with and without glenohumeral osteoarthritis in five variables (age, scapular length, glenoid width, glenopolar angle, and α angle). Scapulae with arthrosis were up to 10 mm shorter and 1 mm narrower with an 8° smaller α angle in comparison to those without arthrosis. Individuals with glenohumeral osteoarthritis were a mean of 7 years older than those without, and glenohumeral osteoarthritis was observed in 21% of females versus 11% of males, although these differences were not statistically significant (Table 4). The chi-square test was applied to evaluate the relationship between glenoid anteversion or retroversion and glenohumeral osteoarthritis development, finding no such association (X^2^ = 1.46, df = 2; p = 0.481).

**Table 4:** Bivariate analysis as a function of glenohumeral osteoarthritis. Student’s t-test.

VARIABLE	GLENOHUMERAL OSTEOARTHRITIS (MEAN)	GLENOHUMERAL OSTEOARTHRITIS (SD)	GLENOHUMERAL OSTEOARTHRITIS (MEAN)	GLENOHUMERAL OSTEOARTHRITIS (SD)	SIGNIFICANCE
AGE	76.09	8.25	69.7	12.02	0.003*
A	135.36	20.94	145.85	13.76	0.004*
B	96.58	7.38	97.39	8.06	0.55
C	31.77	2.34	32.79	3.12	0.116
D	23.11	2.05	24.67	2.71	0.006*
E	45.72	4.37	43.01	5.18	0.018*
G	26.45	2.14	27.07	2.49	0.244
a	60.22	4.20	59.20	6.22	0.447
b	63.22	7.71	65.70	7.71	0.166
α	102.26	16.20	110.26	15.62	0.019*
β	128.15	12.50	123.76	12.26	0.100

P ≤ 0.05

### Multivariate analysis

#### Sex

Scapular width, glenoid height, measurement “a”, measurement “b”, and β angle, which were statistically significant in bivariate analysis, were entered in the multivariate model alongside glenoid height, which was close to significant (p = 0.057). The model proposed to predict the male sex is expressed as follows:

$$P\left(male=1\right)=\frac{1}{1+e^{-\left(-57.9111+0.350*B+0.283*C+0.249*b+0.166*a+-0.100*\beta \right)}}$$

Application of this formula correctly classified the sex of 95% of the individuals under study, including 98.3% of the males and 92.1% of the females. The model explained 64.4% of the variance according to Cox and Snell’s R2 and 84.6% according to Nagelkerke’s R2. Individuals with higher scapular width, glenoid height, and “a” and “b” measurements had a greater likelihood of being male.

#### Degenerative changes

The predictive value of “degenerative changes” on the glenoid joint surface was evaluated as above, adjusting the model for age, scapular length, glenoid width, glenopolar angle, and α angle. The proportion of glenohumeral osteoarthritis variance explained by this model was 7.2% according to Cox and Snell’s R2 and 12.0% according to Nagelkerke’s R2, indicating that the development of glenohumeral osteoarthritis is not directly influenced by these study variables.

## Discussion

The first step in identifying individuals from bone remains is to estimate the sex, given the difference between sexes in the formulas employed to estimate age and stature. Various anthropological studies have been published on sex estimation in Spanish populations [9, 26, 27], and research has been conducted on the sexual dimorphism of the scapula in other populations (Table 5) as Hamann-Todd collection, in a sample of american individuals of late 19th–early 20th century [4] or Italian population of 21^st^ Century [7]. Bone remains from a contemporary Granada population were investigated in 1998 by Alemán et al. to explore the usefulness of different bones for sex estimation, including the scapula [22]. In the present anthropometric study of scapulae in bone remains from a different sample of the same collection, significant differences were found between the sexes in scapula and glenoid size, as previously reported [4, 10], with scapulae generally having larger bone structures in males.

**Table 5:** Bibliographic review of sex estimation based on scapular anthropometry. M: male, F: Female.

STUDIES	INDIVIDUALS	GEOGRAPHIC ORIGIN	CHRONOLOGY	GENDER	AGE (years)	METHODOLOGY	REPORTED OUTCOMES
Alemán et al., 1998	95	Granada	20st Century	45 M 50 F		Analogic anthropometric measurement	Two-variable discriminant function (maximum scapular length and scapular spine length). Accuracy of 92.45%
Ozer et al., 2006	93	Dilkaya medieval collection	Medieval	47 M 46 F		Analogic anthropometric measurement	Maximum scapular breadth 94.8% accuracy
Dabbs et al., 2010	804	American individuals	Late 19th–early 20th century	496 M 308 F	19–93	Analogic anthropometric measurement	Five-variable discriminant function accuracy of 92.5%
Papaioannou et al., 2012	147	Greek population	20st Century	81 M 66 F	Mean 68	Analogic anthropometric measurement	Length of the scapula 91.2% accuracy Two-variable discriminant function accuracy of 95,9% (maximum length of the spine and glenoid cavity breadth)
*Giurazza et al*., *2013*	200	Italian population	21st Century	100 M 100 F	42–86	CT-scan measurement	Two-variable discriminant function accuracy of 88%
Peckmann et al., 2016	114	General cemetery of Santiago of Chile	20st Century	58 M 56 F	17–85	Analogic anthropometric measurement	Two-variable discriminant function (length of the glenoid cavity and breadth of the glenoid cavity) accuracy of 85.6–94.8% in indigenous Guatemalan populations, 85.8–88.3% in South African populations, 95.7% in contemporary American populations, 91.25% in Italian, 94.8% in Anatolian, 96.8% in Japanese, and 80.7–86.0% in Chilean populations
Torimitsu et al., 2016	218	Japanese	21st Century	109 M 109 F	M 22–90 (56.72) F 23–91 (56.48)	CT-scan measurement	Three-variable discriminant function (Left maximum scapula height, left maximum length of the spine and left glenoid cavity breadth) accuracy of 93.1%
*Zhang K et al*., *2016*	414	West China Hospital of Sichuan University	20st Century	224 M 190 F	24–78	Analogic anthropometric measurement	Seven-variable discriminant functions accuracy rate of 86.7%.
*Peckmann et al*., *2017*	191	Thailand	20st Century	95 M 96 F	19–96	Analogic anthropometric measurement	Two-variable discriminant functions (Breadth of glenoid cavity and length of glenoid cavity of the scapula) accuracy rate of 83% to 88%.
*Ali et*.*al*, *2018*	290			184 M 106 F	18–101	CT-scan measurement	Two-variable discriminant function equation (anatomical breadth and anatomical length of the scapula) 94.5% accuracy.

In 2012, Papaioannou et al. [10] described the length of the scapula as the most accurate single measurement for sex estimation, offering an accuracy of 91.2%. However, Özer et al. [12] found the positive predictive value of scapular length to be only 82.9% in an Anatolian population. Both studies found that glenoid height had a predictive value of up to 90%. In 2016, Torimitsu et al. [11] observed that sexual dimorphism was more strongly associated with glenoid height than with glenoid width, although other researchers reached the opposite conclusion [28].

Much greater accuracy can be achieved with a combination of multiple anthropometric parameters rather than a single parameter, although the complete scapula is usually required [4, 10, 12]. In their study of the “Cretan collection”, Papaioannou et al. (2012) obtained a differentiation function for sex estimation based on glenoid fossa width and maximum scapular spine length that yielded an accuracy of 95.9% [10]. A formula that used the height and width of the glenoid fossa, proposed by Peckmann (2016), demonstrated an accuracy of 85.6–94.8% in indigenous Guatemalan populations, 85.8–88.3% in South African populations, 95.7% in contemporary American populations, 91.25% in Italian, 94.8% in Anatolian, 96.8% in Japanese, and 80.7–86.0% in Chilean populations [28]. In their 1998 study, Alemán et al. developed a differentiation function based on the maximum scapular length and scapular spine length, achieving an accuracy of 92.45% [22].

We propose an alternative function that considers the scapular width, glenoid width, measurement “a”, measurement “b”, and β angle. The calculation does not require the whole scapula to be available, only its upper external segment, and it demonstrated 95% accuracy, correctly classifying the male sex in 98.3% of cases, one of the highest percentages published to date.

Few publications (Table 6) have analysed the association between anthropometric parameters of the scapula and glenohumeral osteoarthritis [18, 19, 29]. The prevalence of glenohumeral osteoarthritis was 16.6% in the present series, very close to the prevalence observed in Japanese and Korean populations by imaging studies using the Samilson-Prieto glenohumeral osteoarthritis grade classification [30], which was 17–19%, being bilateral in 3.1–7.7% [31, 32]. In the present series, all cases of glenohumeral osteoarthritis were in over-60-year-olds, and 69.2% were in females, although the difference between sexes was not statistically significant. In the same line, a study of 345 Japanese individuals reported greater prevalence in over-60-year-olds and females [33]. In 1987, Chard et al. described a prevalence of 9.5% among over-60-year-olds in England [34].

**Table 6:** Bibliographic review of glenohumeral osteoarthritis and scapular anthropometry association. M: male, F: Female. CSA: Critical shoulder angle. GHOA: Glenohumeral osteoarthritis, CTA: Cuff tear arthropathy, NS: Normal shoulder, RCT: rotator cuff tear.

STUDIES	INDIVIDUALS	GEOGRAPHIC ORIGINS	CHRONOLOGY	GENDER	AGE (YEARS)	METHODOLOGY	REPORTED OUTCOMES
Viehöfer et al, 2016 [35]		Switzerland				Biomechanical model was used to simulate and measure the joint reaction forces during abduction in the scapular plane for a CSA found in normal and a CSA found in osteoarthritic shoulders	Reduction of the CSA to that typical for patients presenting with osteoarthritis (i.e., a CSA of 28˚) leads to an increase in the magnitude of the net joint reaction force, with a maximal difference of 26 N (8.5%)
Heuberer, et al., 2017	1000	Austria	21st Century	441 M 559 F	39–86	CSA, acromion index and lateral acromion angle measured on anteroposterior radiographs	Two-variable discriminant function (CSA and age) predict five shoulder pathologies of 1000 patients with an accuracy of 67.7%
Miswan, et al., 2017	237	Malasya	21st Century			Radiographs measurement	Changes in acromioglenoid angle is associated with high prevalence of RCT and GHOA
Shinagawa, et al., 2018	295	Japan	21st Century	140 M 155 F	Mean 67 (50–89)	CSAs measured on anteroposterior radiographs	Multivariable analysis showed that greater CSAs significantly increased the risk of rotator cuff tears, with an odds ratio of 1.08 per degree.
Van Parys et al., 2021	205	United Arab Emirates	21st Century	98 M 107 F		Case control study of 3D CT scan images. 49 with GHOA, 48 with CTA, and 108 in NS the center of the glenoid circle and several points at the coracoid, acromion, and glenoid were determined.	The acromial part of the complex was turned more posteriorly in both pathologic groups. Furthermore, we found the coracoacromial complex to be more cranial to the glenoid center in the GHOA group.
Verhaeg et al., 2021	227	Belgium	21st Century	42% M 58% F	66	Case control study of 3D CT scan images of 110 healthy shoulder patients and 117 shoulder osteoarthritis patients.	Patients with osteoarthritis had a significantly lower CSA, posterior acromion-scapular plane angle, coracoid-posterior acromion angle, and fulcrum axis ratio osteoarthritis.

Multiple risk factors have been described for the development of glenohumeral osteoarthritis, including age, sex, obesity, previous fractures, occupation, rotator cuff lesion, arthritis, avascular necrosis [20, 36–38], and scapular morphology [39]. The relationship of scapular morphology with the development of shoulder and glenohumeral joint diseases is a hot topic in traumatology [39, 40]. Van Parys et al. associated the presence of glenohumeral osteoarthritis with a smaller “scapular arch”, defined as the angle between the coracoid process and most posterior point of the acromion, with its center in the glenoid fossa. It has been reported that this angle, designated the α angle in the present study, appears to be smaller in individuals with glenohumeral osteoarthritis [18, 19]. It was significantly reduced in the present series of individuals with glenohumeral osteoarthritis, who showed a mean difference of 8° *versus* those without this disease. According to Van Parys et al., the coracoacromial arch is more centered on the glenoid fossa in individuals with glenohumeral osteoarthritis; this creates a larger subacromial space with lesser containment of the humeral head at craniocaudal level, thereby increasing compressive forces from the rotator cuff with the consequent joint reaction and glenohumeral osteoarthritis development [18].

Glenoid fossa in retroversion were observed in 62.4% of the present series, but none exceeded 25°, and no significant differences were observed in glenoid version between individuals with and without glenohumeral osteoarthritis, as also reported by Verhaegen in 2021 [19]. Anthropometric variables with significant or close-to-significant associations with shoulder glenohumeral osteoarthritis in bivariate analyses (age, scapular length, glenoid width, glenopolar angle, and α angle) were not maintained in the predictive multivariate model for glenohumeral osteoarthritis.

Study limitations include that information was gathered from a single population, representing a selection bias. It was also not possible to analyze environmental or lifestyle factors, which may influence scapular development. It should also be borne in mind that the estimation of glenohumeral osteoarthritis derived from bone remains likely underestimates its presence, due to the absence of articular cartilage. Finally, because a single observer performed the measurements in only one stage; no data were obtained on their reproducibility.

## Conclusions

A sex estimation capacity of 95% was obtained using a novel discrimination function based on bone remains of the upper-external segment of the scapula; it considers scapular width, glenoid height, measurement “a”, measurement “b”, and β angle.

Although glenohumeral osteoarthritis is significantly associated with a smaller α angle, its development does not appear to be influenced by glenoid version. It proved impossible to establish a predictive model for glenohumeral osteoarthritis based on the anthropometric parameters studied in this population.

## Supporting information

S1 Dataset Data base. (XLSX)
